# Platypnea-orthodeoxia syndrome in the right lateral decubitus position: a case report

**DOI:** 10.1186/s13256-017-1267-6

**Published:** 2017-04-12

**Authors:** Ippei Tsuzuki, Kamon Iigaya, Takashi Matsubara, Shunsuke Takagi, Taku Inohara, Yasuyuki Ohgino, Toshio Imafuku

**Affiliations:** 1 Department of Internal Medicine, Hiratsuka city Hospital, 1-19-1 Minamihara, Hiratsuka city, Kanagawa 254-0065 Japan; 2 Department of Cardiology, Hiratsuka city Hospital, 1-19-1 Minamihara, Hiratsuka city, Kanagawa 254-0065 Japan

**Keywords:** Case report, Platypnea-orthodeoxia syndrome, Hemidiaphragmatic elevation, Giant liver cyst, Pacemaker lead, Patent foramen ovale

## Abstract

**Background:**

Platypnea-orthodeoxia syndrome is a rare syndrome characterized by dyspnea and hypoxia when the patient is sitting or standing. Here we report a case of platypnea-orthodeoxia syndrome caused by a right hemidiaphragmatic elevation with giant liver cyst that triggered a right-to-left shunt through the patent foramen ovale. This case report is the first presentation of a case secondary to hemidiaphragmatic elevation with giant liver cyst. In addition to this, a malposition of the pacemaker lead could be associated with platypnea-orthodeoxia syndrome in this case.

**Case presentation:**

A 91-year-old Japanese woman presented to our hospital with hypoxia of unknown origin. Severe hypoxia and cyanosis were observed only in the right lateral decubitus position. A chest X-ray and computed tomography scan revealed right hemidiaphragmatic elevation, which was probably compressing the right atrium. A transesophageal echocardiogram showed a compressed right atrium and shunt blood flow in both directions: from the left to the right atrium and vice versa. The shunt flow was exacerbated by postural changes from the left to the right lateral decubitus. A transesophageal echocardiogram also confirmed compression of the right atrium due to giant liver cyst and a malposition of the pacemaker lead abnormally placed in the left atrium through patent foramen ovale. We concluded that the cause of hypoxia was platypnea-orthodeoxia syndrome with right-to-left interatrial shunt through patent foramen ovale. Surgical closure of patent foramen ovale was not performed due to the age of our patient, surgical difficulties, and failure to obtain informed consent. For these reasons she was discharged after receiving medical advice about her posture.

**Conclusions:**

Platypnea-orthodeoxia syndrome is rare and difficult to diagnose. The present case suggests that hypoxia due to postural changes should be considered a differential diagnosis of platypnea-orthodeoxia syndrome.

## Background

Platypnea-orthodeoxia syndrome (POS) is a rare syndrome characterized by dyspnea and hypoxia when the patient is sitting or standing. These symptoms are relieved when the patient is recumbent. POS was first described in 1949 by Burchell *et al*. [1].

Here we report a case of POS caused by a right hemidiaphragmatic elevation that was triggered by a giant liver cyst that triggered an increase in atrial right-to-left shunt in the right lateral decubitus position through the patent foramen ovale (PFO), which was probably caused by a malposition of a pacemaker lead through the PFO.

## Case presentation

A 91-year-old Japanese woman presented to our hospital with hypoxia of unknown origin. She had a medical history of hypertension and atrial fibrillation. Eight years before, when she was 82, an advanced atrioventricular block was diagnosed after a syncopal episode and she received a permanent dual chamber pacemaker implantation. She underwent coronary computed tomography (CT) and radioisotope examination at this time, but there was no evidence of cardiomyopathy or of ischemic heart disease. The tip of the pacemaker lead was observed to pass through a PFO at the left ventricular apex; she received a follow-up examination because of no serious adverse event. She had no history of lung disease or tobacco smoking. She had no subjective symptoms associated with cardiovascular disease after pacemaker implantation. There was no evidence of a decline in oxygen saturation levels at that time, which were measured by pulse oximetry.

Her physical examination findings on admission were as follows: clear level of consciousness and severe hypoxia and cyanosis observed only in the right lateral decubitus position. These symptoms were immediately improved in the left lateral decubitus, the supine, and the upright position. Arterial blood gas values were consistent with her symptoms. Partial arterial oxygen pressure (PaO_2_) and arterial oxygen saturation (SaO_2_) decreased only in the right lateral decubitus position (Table 1). These observations were reproducible. There was no evidence of lung rales or cardiac murmur.

**Table 1:** Arterial blood gas value with room air

Position	Upright	Right lateral	Left lateral	Supine
PaO_2_ (mmHg)	55.3	45	76.2	54.7
PaCO_2_ (mmHg)	32.1	28.4	29.8	32.1
SaO_2_ (%)	90.1	83.6	95.4	90.1

*PaCO*_*2*_ partial arterial carbon dioxide pressure, *PaO*_*2*_ partial arterial oxygen pressure, *SaO*_*2*_ arterial oxygen saturation

Laboratory tests, including complete blood count, thyroid function tests, and D-dimers were almost normal. A chest X-ray showed a significant elevation of her right diaphragmatic dome.

**Fig. 1:**
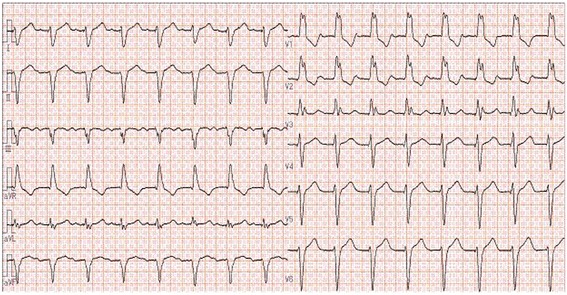
Twelve-lead electrocardiogram. Note the right bundle branch block on ventricular pacing beats

**Fig. 2:**
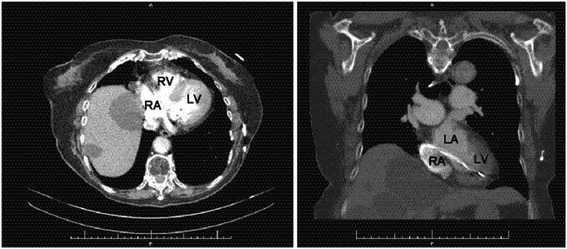
Contrast-enhanced computed tomography showed compression of the right atrium because of a giant liver cyst and the pacemaker lead wire running from the right atrium to the left ventricle. *LA* left atrium, *LV* left ventricle, *RA* right atrium, *RV* right ventricle

An electrocardiogram (ECG) showed an atrium-sensing, ventricular-pacing rhythm with a complete right bundle branch block (RBBB) pattern (Fig. 1). In general, when the pacemaker lead is set on the right ventricle correctly, the pacemaker first stimulates the right ventricle, and next the left ventricle. Because of this, the expected ECG pattern with right ventricle pacing should show a left bundle branch block (LBBB) pattern. A RBBB pattern suggests that the pacemaker first stimulates the left ventricle, and next the right one. Only this ECG pattern could indicate the possibility of inadvertent left ventricle pacing. This patient’s ECG pattern also suggested left ventricle pacing. However, we could not assess a malposition of the pacemaker lead from only an ECG pattern because sometimes we find a RBBB pattern although the pacemaker lead is set correctly. Contrast-enhanced CT excluded the diagnosis of cerebral infarction, pulmonary embolism, pneumonia, and lung parenchymal abnormalities and revealed giant liver cysts and compression of the right atrium by one of cysts (Fig. 2). A chest X-ray and CT scan revealed right hemidiaphragmatic elevation that was probably compressing the right atrium. This finding was suspected in the case of right hemidiaphragmatic paralysis [2]. A transthoracic echocardiogram (TTE) revealed concentric left ventricular hypertrophy, left atrial dilatation, and compression of the right atrium due to the giant liver cyst. In addition, the pacemaker lead probably ran abnormally to the left atrium and ventricle through PFO, but shunt flow across the PFO on color Doppler image was not apparently observed. Pulmonary perfusion imaging with ^99m^Tc-macroaggregated albumin (MAA) was performed in the right lateral decubitus position. MAA accumulated in her kidneys and brain, which suggested the presence of a right-to-left shunt. The estimated shunt ratio was 26.3%.

**Fig. 3:**
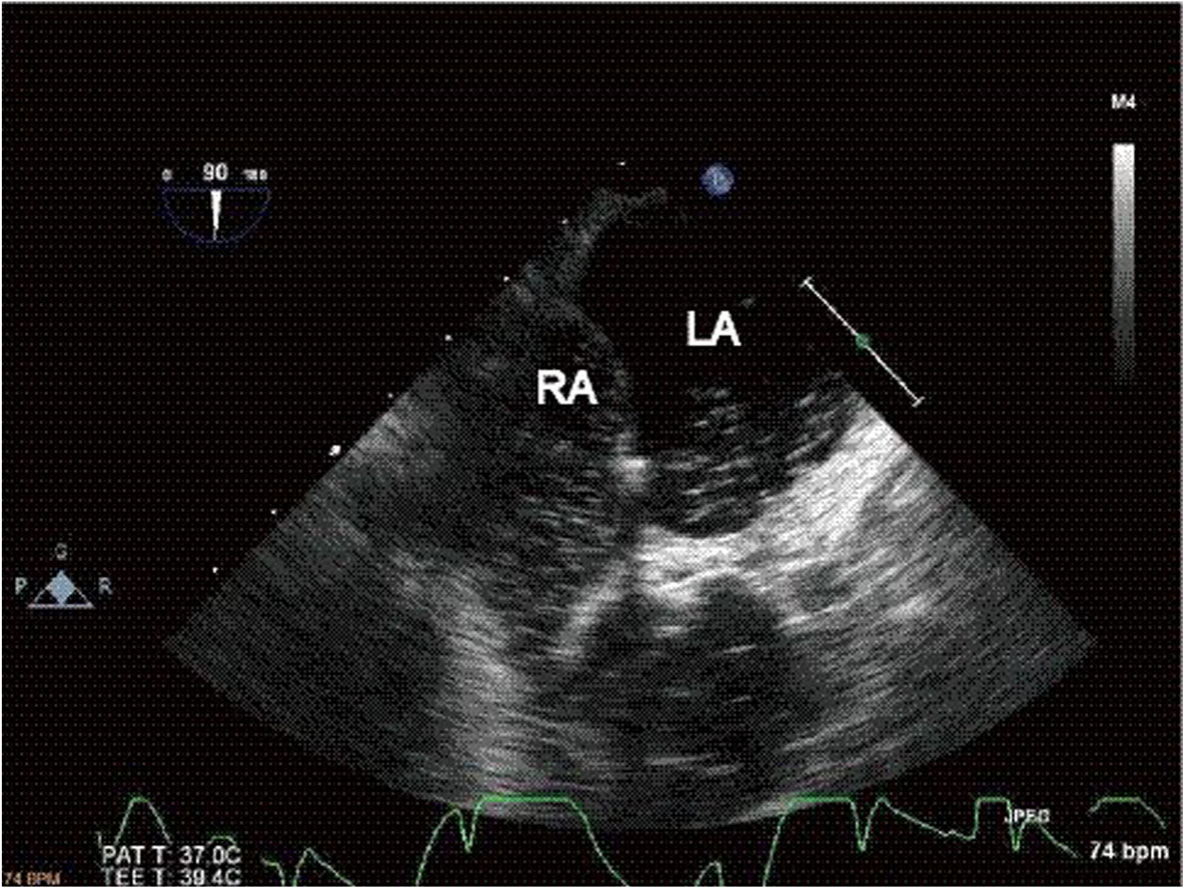
The bubble contrast study showed the presence of right-to-left shunt through patent foramen ovale. *LA* left atrium, *RA* right atrium

**Fig. 4:**
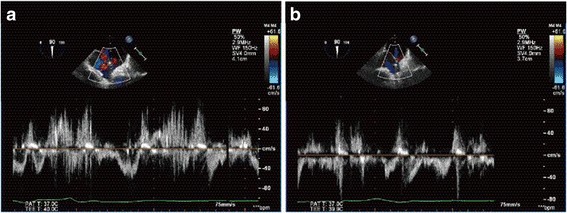
Pulse Doppler demonstrated increased right-to-left shunt in the right lateral decubitus position (**a**) compared with the left lateral decubitus position (**b**)

**Fig. 5:**
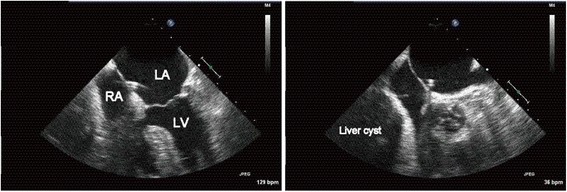
Transesophageal echocardiogram revealed that the giant liver cyst compressed the right atrium and the pacemaker lead was abnormally running to the left atrium through the patent foramen ovale. *LA* left atrium, *LV* left ventricle, *RA* right atrium

A transesophageal echocardiogram (TEE) with intravenously administered agitated saline contrast solution was performed in both right and left lateral decubitus (Fig. 3). TEE showed a compressed right atrium and shunt blood flow in both directions: from the left to the right atrium and vice versa. The shunt flow was exacerbated by postural changes from the left to the right lateral decubitus (Fig. 4). TEE also confirmed that the giant liver cyst was compressing the right atrium and the pacemaker lead was abnormally running to the left atrium through PFO (Fig. 5).

Considering these results, we concluded that the cause of hypoxia was POS with right-to-left interatrial shunt through PFO. POS can be treated with the closure of the interatrial shunt by thoracotomy or percutaneous cardiac catheterization [3]. Surgical closure of PFO was not performed due to the age of our patient, surgical difficulties, and failure to obtain informed consent. For these reasons she was discharged after receiving medical advice about her posture.

## Discussion

The most important clue for diagnosis of POS is the emergence of hypoxia with postural changes. Several previous cases have demonstrated that the increased shunt flow across PFO or atrial septal defect (ASD) with posture changes from supine to upright induce hypoxia [4]. The standing upright position can directly change the anatomical route from the vena cava to an intracardiac defect in a straight line, which increases right-to-left shunt [3]. However, some cases of POS where symptoms were exacerbated in the lateral decubitus position were reported [5, 6]. In the present case, hypoxia was recognized only in the right lateral decubitus but not in the upright position. As it is the same concept of POS, which induced hypoxia by an increase in right-to-left shunt with posture change, we discuss the pathophysiology of the present case starting from the mechanism of POS.

POS is caused by the coexistence of anatomical and functional abnormalities [3]. Anatomical abnormalities include ASD, PFO, or fenestrated atrial septal aneurysm. However, the presence of only these anatomical factors cannot induce POS because they usually cause left-to-right shunting. In addition, functional factors, such as right hemidiaphragmatic paralysis, pericardial effusion, constrictive pericarditis, emphysema, arteriovenous malformation, pneumonectomy, liver cirrhosis, aortic aneurysm, or elongation changes of shunt flow can determine POS [7]. Many previous reports indicated that POS can occur for various reasons.

There are two theories on the occurrence of right-to-left shunt-induced POS without pulmonary hypertension. One theory suggests a hemodynamic phenomenon with an interatrial pressure gradient, and the other theory is derived from selective blood flow from the vena cava to the left atrium passing through an intercardiac defect [8]. The latter mechanism is often associated with a straight connection from the vena cava to the left atrium, which generates direct shunting flow.

Because of the lower sensitivity of TTE for small shunts, the prevalence of PFO observed by contrast TTE in the general population was reported to be lower than that observed by TEE (14.9% versus 24.3%) [9]; this indicates that only TTE can fail the diagnosis of ASD or PFO. POS caused by PFO can be clearly demonstrated by the technique of contrast TEE [10]. Interatrial right-to-left shunt and the giant liver cyst compressing the right atrium were revealed by TEE but not TTE in this case.

POS due to intracardiac shunting in patients with liver disease is quite rare [5]. Patakas *et al*. [11] reported a case of POS by large hydatid cysts compressing the right heart chambers. Mohamad *et al*. [5] reported a case of POS resulting from a complication of partial liver resection. In these two cases, the compression of the right atrium by abnormal liver lesions might have been associated with POS.

Our patient also had two factors leading to POS: PFO and abnormal geometry. A large liver cyst may have contributed to this abnormality. Although she had giant liver cysts, she had no evidence of liver cirrhosis or hepatopulmonary syndrome. There were no other explanations for her POS, such as pericardial effusion, constrictive pericarditis, pulmonary disease, ileus, or aortic events. Two factors may have led to abnormal right-to-left shunting in this case. The first mechanism is related to compression of the right atrium by the giant liver cyst, resulting in a relative increase of the right atrial pressure causing right-to-left shunt. The second mechanism is related to the giant liver cyst deforming the right-sided heart chambers and causing the development of direct shunt flow between her vena cava and the left atrium across PFO. In the right decubitus, her heart was in close contact with the giant liver cyst. In all other postural positions, her heart did not have such tight contact with the liver cyst, minimizing the effects of compression.

As previously stated, a chest X-ray and CT scan showed right hemidiaphragmatic elevation in this case. There are various etiologies for it such as hemidiaphragmatic paralysis, hepatomegaly, eventration of the diaphragm, and diaphragmatic hernia. In this case, hepatomegaly with multiple liver cysts seemed to trigger hemidiaphragmatic elevation. A few case studies reported POS with hemidiaphragmatic elevation, especially triggered by hemidiaphragmatic paralysis [12–15], but this case report is the first presentation of a case secondary to hemidiaphragmatic elevation with giant liver cyst. Whatever caused hemidiaphragmatic elevation, it may have led to the compression of the right atrium, and change of anatomical relation of the vena cava and the left atrium across PFO, resulting in POS. However, the exact mechanism causing POS by hemidiaphragmatic elevation is still unknown [16].

One more factor in this case can be correlated with POS; during pacemaker implantation, the lead was accidentally misplaced through PFO. The pacemaker lead was assumed to stretch the circumference of PFO in the right lateral decubitus position, and increasing the area of PFO might increase the right-to-left shunt flow.

There are previous reports where surgical closure of ASD or PFO were successfully performed [17–19]; however, percutaneous transcatheter closure devices have recently come into use because of their reduced invasiveness and are easy and safe compared with open surgery [8, 20].

Although withdrawal of the pacemaker and volume reduction therapy for the giant liver cyst should have been performed to decrease the intracardiac right-to-left shunt flow, no therapy was performed in this case due to procedural risks. Surgical closure of PFO was not performed due to the age of our patient, surgical difficulties, and failure to obtain informed consent. For these reasons our patient was discharged after receiving medical advice about her posture.

## Conclusions

POS is rare and difficult to diagnose. The present case suggests that hypoxia caused by posture changes in patients should be considered a differential diagnosis of POS.

## Abbreviations

ASD: Atrial septal defect
CT: Computed tomography
ECG: Electrocardiogram
LBBB: Left bundle branch block
MAA: Macroaggregated albumin
PaO_2_: Partial arterial oxygen pressure
PFO: Patent foramen ovale
POS: Platypnea-orthodeoxia syndrome
RBBB: Right bundle branch block
SaO_2_: Arterial oxygen saturation
TEE: Transesophageal echocardiogram
TTE: Transthoracic echocardiogram
